# Supplementary material for: Reconsidering the Tolerable Upper Levels of Zinc Intake among Infants and Young Children: A Systematic Review of the Available Evidence
Source: Nutrients. 2022 May 5;14(9):1938. doi: 10.3390/nu14091938 (PMC9102402; doi:10.3390/nu14091938)
Supplement: Supplementary file 1 [file nutrients-14-01938-s001.zip › nutrients-1679471-supplementary.pdf]

Reconsidering the tolerable upper levels of zinc intake among infants and young children: A systematic review of the available evidence

Sara Wuehler[1*], Daniel Lopez de Romaña[1], Demewoz Haile[2,3], Christine M. McDonald[4,5]and Kenneth H. Brown[2*]

1 Nutrition International, Canada
2 Department of Nutrition and Institute for Global Nutrition, University of California Davis, CA USA
3 Institute for Health Metrics and Evaluation, University of Washington, Seattle, Washington USA
4 Department of Pediatrics, University of California, San Francisco, CA. USA
5 International Zinc Nutrition Consultative Group, Oakland, CA
* Correspondence: S.W.* swuehler@nutritionintl.org; D.LdR. dlopezderomana@nutritionintl.org; D.H. dewolde@ucdavis.edu; C.M. Christine.Mcdonald@ucsf.edu; K.B.* khbrown@ucdavis.edu

Supplementary Materials
Supplemental Tables

Table S1. Studies and comparisons with reported serum or plasma copper outcomes in response to additional zinc supplementation or fortification, by author.

Study	Year	Country	Sample size analyzed	Characteristics of subjects	Initial age	Duration, m	Schedule	Intervention (All controls are same without the added zinc)	Form of zinc	Dose, mg[1]	Actual dose, mg/d[1]	Effect of zinc§
Baqui [1]	2005	Bangladesh	80	Healthy infants	6 m	6	Weekly	Zn + B_2 supplement	Acetate	20	2.9	±
Baqui [1]	2005	Bangladesh	82	Healthy infants	6 m	6	Weekly	Zn + Fe + B_2 supplement	Acetate	20	2.9	±
Bertinato [2]	2013	Canada	20	Healthy children, male	6-8 y	4	Daily	Zn supplement	Gluconate	5	5	±
Bertinato [2]	2013	Canada	19	Healthy children, male	6-8 y	4	Daily	Zn supplement	Gluconate	10	10	±
Bertinato [2]	2013	Canada	18	Healthy children, male	6-8 y	4	Daily	Zn supplement	Gluconate	15	15	±
Bhandari [3]	2002	India	930	Healthy infants and children	6-30 m	4	Daily	Zn supplement	Gluconate	10 for < 12 m-old & 20 for ≥12 m-old	10 for < 12 m-old & 20 for ≥12 m-old	↓
Brooks [4]	2005	Bangladesh	636	Healthy infants	1-12 m	10	Weekly	Zn supplement	Acetate	70	10	[±]*

Brown [5]	2007	Peru	144	Healthy infants	6-8 m	6	Daily	Zn + other MN supplement, + Fe fortified food	Sulfate	3	2.3	±
Brown [5]	2007	Peru	147	Healthy infants	6-8 m	6	Daily	Zn + Fe fortified complementary food + MN supplement (no zinc)	Sulfate	3	3.3	↓ [±]*
Caulfield [6]	2013	Peru	159	Healthy infants	4-6 m	18	Daily	Zn + Fe + Cu supplement	Sulfate	10	10	±
Hambidge [7]	1979	USA	58	Healthy children	33-90 m	9	Daily	Zn fortified breakfast cereal (control, no fortificant)	Oxide	3 (difference from control; 9.3) Control: 6.3	»3	↓ [±]*
Heinig [8]	2006	USA	19	Healthy infants	4 m	6	Daily	Zn supplement	Gluconate or Sulfate	5	5	↓
Lind [9]	2003	Indonesia	277	Healthy infants	6 m	6	Daily	Zn supplement	Sulfate	10	10	±
Lind [9]	2003	Indonesia	272	Healthy infants	6 m	6	Daily	Zn + Fe supplement	Sulfate	10	10	±
Radhakrishna [10]	2013	India	68	Healthy infants	4 m	14 (12 with suppl)	Daily	Intervention Zn + B2 supplement	Sulfate	5	5	±
Rosado [11]	2006	Mexico	187	Healthy children with environmental lead exposure	6-7 y	6	5d/wk	Zn supplement	Oxide	30	21.4	±
Rosado [11]	2006	Mexico	198	Healthy children with environmental lead exposure	6-7 y	6	5d/wk	Fe + Zn supplement	Oxide	30	21.4	±
Ruz [12]	1997	Chile	69	Healthy children	27-50 m	6 biochemical	Daily	Zn supplement	Sulfate	10	10	±
Sazawal [13]	2004	India	110	Healthy infants	5-12 m	4	Daily	Zn supplement + multi-vitamins	Gluconate	10	10	±
Tielsch [14]	2007	Nepal	197	Healthy infants and children	1-35 m	12	Daily	Zn supplement	Sulfate	5 for 1-11 m-old, 10 for 12-35 m-old	5 for 1-11 m-old, 10 for 12-35 m-old	±

Walravens [15]	1976	USA	68	Healthy infants	4-6 d	6	Daily	Zn + Fe fortified formula	Sulfate	4 more than (1.8) Control: 1.8	~4	↑
Walravens [16]	1983	USA	40	Zn deficient children	2-6 y	12	Daily	Zn supplement	Sulfate	10 (average 4.2/d based on compliance)	10	±
Wuehler [17]	2008	Ecuador	110	Healthy children	12-30 m	6	Daily	Zn supplement	Sulfate	10	10	±

*In brackets is the effect as reported by the original study. ↑ or ↓ indicates the final concentration is higher or lower, respectively, in the zinc intervention group compared to the non-zinc comparison; ± indicates no reported difference between zinc and no-zinc interventions.

[1] All control groups received the same as the intervention except the dose of zinc listed

Table S2. Studies and comparisons with reported serum or plasma ceruloplasmin outcomes in response to additional zinc supplementation or fortification, by author.

Study	Year of publication	Country	Sample size analyzed	Characteristics of subjects	Initial age	Duration, m	Schedule	Intervention (All controls are same without the added zinc)	Form of zinc	Dose[1], mg	Actual dose[1], mg/d	Effect of zinc§
Bertinato [2]	2013	Canada	20	Healthy children, male	6-8 y	4	Daily	Zn supplement	Gluconate	5	5	±
Bertinato [2]	2013	Canada	19	Healthy children, male	6-8 y	4	Daily	Zn supplement	Gluconate	10	10	±
Bertinato [2]	2013	Canada	18	Healthy children, male	6-8 y	4	Daily	Zn supplement	Gluconate	15	15	±
De Brito [18]	2014	Brazil	30	Healthy children	8-9 y	3	Daily	Zn added to milk or juice	Sulfate	10	10	↓ [±]*
Wuehler [17]	2008	Ecuador	110	Healthy children	12-30 m	6	Daily	Zn supplement	Sulfate	10	10	±

*In brackets is the effect as reported by the original study. ↑ or ↓ indicates the final concentration is higher or lower, respectively, in the zinc intervention group compared to the non-zinc comparison; ± indicates no reported difference between zinc and no-zinc interventions.

[1] All control groups received the same as the intervention except the dose of zinc listed

Table S3. Studies and comparisons with reported erythrocyte superoxide dismutase (ESOD) outcomes in response to additional zinc supplementation or fortification, by author.

**Study**	**Year**	**Country**	**Sample size analyzed**	**Characteristics of subjects**	**Initial age**	**Duration, m**	**Schedule**	**Intervention (All controls are same without the added zinc)**	**Form of zinc**	**Dose[1], mg**	**Actual dose[1], mg/d**	**Effect of zinc§**
Bates [19]	1993	Gambia	106	Healthy infants and children	6-28m	2	Bi-Weekly	Zn supplement	Acetate then gluconate	70	20	±
Bertinato [2]	2013	Canada	18	Healthy children, male	6-8 y	4	Daily	Zn supplement	Gluconate	15	15	±
Bertinato [2]	2013	Canada	20	Healthy children, male	6-8 y	4	Daily	Zn supplement	Gluconate	5	5	±
Bertinato [2]	2013	Canada	19	Healthy children, male	6-8 y	4	Daily	Zn supplement	Gluconate	10	10	↓ [±]*
Heinig [8]	2006	USA	19	Healthy infants	4 m	6	Daily	Zn supplement	Gluconate or Sulfate	5	5	±
Wuehler [17]	2008	Ecuador	110	Healthy children	12-30 m	6	Daily	Zn supplement	Sulfate	10	10	±

*In brackets is the effect as reported by the original study. ↑ or ↓ indicates the final concentration is higher or lower, respectively, in the zinc intervention group compared to the non-zinc comparison; ± indicates no reported difference between zinc and no-zinc interventions.

[1] All control groups received the same as the intervention except the dose of zinc listed

Table S4. Studies and comparisons with reported serum or plasma ferritin outcomes in response to additional zinc supplementation or fortification, by author.

Study	Year	Country	Sample size analyzed	Characteristics of subjects	Initial age	Duration, m	Schedule	Intervention (All controls are same without the added zinc)	Form of zinc	Dose[1], mg	Actual dose[1], mg/d	Effect of zinc§
Abdollahi [20]	2019	Iran	308	Healthy infants	6-24 m	6	Daily	Zn supplement (program provides Fe)	Sulfate	5	5	±
Alarcon [21]	2004	Peru	213	Anemic infants and children (70-100, moderate anemia)	6-35m	4.1 (18wks)	6d/wk	Fe + Zn supplement	Sulfate	7.6	6.5	↑
Baqui [1]	2005	Bangladesh	77	Healthy infants	6 m	6	Weekly	Zn+ B_2 supplement	Acetate	20	2.9	±
Baqui [1]	2005	Bangladesh	79	Healthy infants	6 m	6	Weekly	Zn + Fe + B_2 supplement	Acetate	20	2.9	±
Bates [19]	1993	Gambia	85	Healthy infants and children	6-28m	2	Bi-Weekly	Zn supplement	Acetate then gluconate	70	20	±
Becquey [22] data from authors	2016	Burkina Faso	169	Healthy infants and children	6-27 m	11	IPZS 10 d every 16 wk (IPZS)	Zn supplement	Sulfate	7	0.9	↓ [±]*
Becquey [22] data from authors	2016	Burkina Faso	165	Healthy infants and children	6-27 m	11	DPZS Daily (DPZS)	Zn supplement	Sulfate	7	7	±
Berger [23]	2006	Vietnam	249	Healthy infants	4-7 m	6	Daily	Zn supplement	Sulfate	10	10	±
Berger [23]	2006	Vietnam	327	Healthy infants	4-7 m	6	Daily	Zn + Fe supplement	Sulfate	10	10	±
Brown [5]	2007	Peru	144	Healthy infants	6-8 m	6	Daily	Zn supplement + Fe fortified porridge	Sulfate	3	2.3	±
Brown [5]	2007	Peru	147	Healthy infants	6-8 m	6	Daily	Zn + Fe fortified complementary food	Sulfate	3	3.3	±
Carter [24]	2018	Tanzania	302	Healthy infants	5-7 wk	19-21 wks	Daily	Zn supplement	Sulfate	5 until blood draw at 6 m of age	5 until blood draw at 6 m of age	±

Carter [24]	2018	Tanzania	291	Healthy infants	5-7 wk	19-21wks	Daily	Zn + MV (no Fe) supplement	Sulfate	5 until blood draw at 6 m of age	5 until blood draw at 6 m of age	±
Caulfield [6]	2013	Peru	170	Healthy infants	4-6 m	18	Daily	Zn + Fe + Cu supplement	Sulfate	10	10	±
De Brito [18]	2014	Brazil	30	Healthy children	8-9 y	3	Daily	Zn added to milk or juice	Sulfate	10	10	±
Dijkhuizen [25]	2001	Indonesia	184	Healthy infants	4 m	6	5 d/wk	Zn supplement	Sulfate	10	7.1	±
Dijkhuizen [25]	2001	Indonesia	164	Healthy infants	4 m	6	5 d/wk	Zn + Fe supplement	Sulfate	10	7.1	↓ [±]*
Fahmida [26]	2007	Indonesia	58	Anemic infants (80% anemia)	3-6 m	6	Daily	Zn supplement	Sulfate	10	10	↓ [±]*
Heinig [8]	2006	USA	19	Healthy infants	4 m	6	Daily	Zn supplement	Gluconate or Sulfate	5	5	±
Lind [9]	2003	Indonesia	277	Healthy infants	6 m	6	Daily	Zn supplement	Sulfate	10	10	±
Lind [9]	2003	Indonesia	272	Healthy infants	6 m	6	Daily	Zn + Fe supplement	Sulfate	10	10	↓
Lopez de Romaña [27]	2005	Peru	22	Stunted and anemic children	3-4 y	1.6	5d/wk	Zn + Fe fortified wheat	Sulfate	3	2.1	±
Lopez de Romaña [27]	2005	Peru	24	Stunted and anemic children	3-4 y	1.6	5d/wk	Zn + Fe fortified wheat	Sulfate	9	6.4	±
Rosado [11]	2006	Mexico	187	Healthy children with environmental lead exposure	6-7 y	6	5d/wk	Zn supplement	Oxide	30	21.4	±
Rosado [11]	2006	Mexico	198	Healthy children with environmental lead exposure	6-7 y	6	5d/wk	Fe + Zn supplement	Oxide	30	21.4	±
Rosado [28]	1997	Mexico	95	Healthy children	18-36 m	12	5d/wk	Zn supplement	Methionine	20	14.3	±
Rosado [28]	1997	Mexico	99	Healthy children	18-36 m	12	5d/wk	Fe + Zn supplement	Methionine	20	14.3	±

Ruz [12]	1997	Chile	68	Healthy children	27-50 m	6	Daily	Intervention; Zn supplement	Sulfate	10	10	±
Soofi [29]	2013	Pakistan	317	Healthy infants	6 m	12	Daily	Zn + Fe + MNP supplement	Gluconate	10	10	±
Tielsch [30]	2006	Nepal	310	Healthy infants and children	1-35 m	12	Daily	Zn + Fe + FA supplement	Sulfate	5 for 1-11 m-old, 10 for 12-35 m-old	5 for 1-11 m-old, 10 for 12-35 m-old	↓
Veenemans [31]	2011	Tanzania	299	Healthy infants and children	6-60 m	8.3	Daily	Zn supplement	Gluconate	10	10	↓
Veenemans [31]	2011	Tanzania	299	Healthy infants and children	6-60 m	8.3	Daily	Zn + Fe + MMN supplement	Gluconate	10	10	↓
Wasantwisut [32]	2006	Thailand	256	Healthy infants, Hb 80+ g/L	4-6 m	6	Daily	Zn + VitC supplement	Sulfate	10	10	±
Wasantwisut [32]	2006	Thailand	256	Healthy infants, Hb 80+ g/L	4-6 m	6	Daily	Zn + Fe + VitC supplement	Sulfate	10	10	↓
Wieringa [33]	2003	Indonesia	91	Healthy infants	4 m	6	5d/wk	Zn supplement	Sulfate	10	7.1	↓ [±]*
Wieringa [33]	2003	Indonesia	77	Healthy infants	4 m	6	5d/wk	Zn + β-carotene supplement	Sulfate	10	7.1	±
Wieringa [33]	2003	Indonesia	88	Healthy infants	4 m	6	5d/wk	Zn + Fe supplement	Sulfate	10	7.1	±
Wuehler [17]	2008	Ecuador	110	Healthy children	12-30 m	6	Daily	Zn supplement	Sulfate	10	10	±
Zlotkin [34]	2003	Ghana	202	Infants and children with mild-moderate anemia	6-18 m	2	Daily	Zn + Fe + VitC supplement	Gluconate	10	10	±

*In brackets is the effect as reported by the original study. ↑ or ↓ indicates the final concentration is higher or lower, respectively, in the zinc intervention group compared to the non-zinc comparison; ± indicates no reported difference between zinc and no-zinc interventions.

[1] All control groups received the same as the intervention except the dose of zinc listed

Table S5. Studies and comparisons with reported hemoglobin outcomes in response to additional zinc supplementation or fortification, by author.

Study	Year	Country	Sample size analyzed	Characteristics of subjects	Initial age	Duration, m	Schedule	Intervention (All controls are same without the added zinc)	Form of zinc	Dose[1], mg	Actual dose[1], mg/d	Effect of zinc§
Alarcon [21]	2004	Peru	213	Anemic infants and children (70-100, moderate anaemia)	6-35m	4.1 (18wks)	6d/wk	Fe + Zn supplement	Sulfate	0.7mg/kg/d	6.5	↑
Baqui [1]	2005	Bangladesh	124	Healthy infants	6 m	6	Weekly	Zn+ B_2 supplement	Acetate	20	2.9	±
Baqui [1]	2005	Bangladesh	125	Healthy infants	6 m	6	Weekly	Zn + Fe + B_2 supplement	Acetate	20	2.9	±
Becquey [22] data from authors	2016	Burkina Faso	169	Healthy infants and children	6-27 m	11	10 d every 16 wk	Zn supplement	Sulfate	7	0.9	±
Becquey [22] data from authors	2016	Burkina Faso	165	Healthy infants and children	6-27 m	11	Daily	Zn supplement	Sulfate	7	7	±
Berger [23]	2006	Vietnam	381	Healthy infants	4-7 m	6	Daily	Zn supplement	Sulfate	10	10	±
Berger [23]	2006	Vietnam	379	Healthy infants	4-7 m	6	Daily	Zn + Fe supplement	Sulfate	10	10	±
Brooks [4]	2005	Bangladesh	636	Healthy infants	1-12 m	10	Weekly	Zn supplement	Acetate	70	10	±
Brown [5]	2007	Peru	144	Healthy infants	6-8 m	6	Daily	Zn supplement + Fe fortified porridge	Sulfate	3	2.3	±
Brown [5]	2007	Peru	147	Healthy infants	6-8 m	6	Daily	Zn + Fe fortified complementary food	Sulfate	3	»3.3	±
Carter [24]	2018	Tanzania	302	Healthy infants	5-7 wk	18	Daily	Zn supplement	Sulfate	5 until 6 m of age then 10	5 until 6 m of age then 10	±
Carter [24]	2018	Tanzania	291	Healthy infants	5-7 wk	18	Daily	Zn + MV (no Fe) supplement	Sulfate	5 until 6 m of age then 10	5 until 6 m of age then 10	±
Caulfield [6]	2013	Peru	209	Healthy infants	4-6 m	18	Daily	Zn + Fe + Cu supplement	Sulfate	10	10	± [↑]*
De Brito [18]	2014	Brazil	30	Healthy children	8-9 y	3	Daily	Zn added to milk or juice	Sulfate	10	10	±
Dijkhuizen [25]	2001	Indonesia	184	Healthy infants	4 m	6	5 d/wk	Zn supplement	Sulfate	10	7.1	±
Dijkhuizen [25]	2001	Indonesia	164	Healthy infants	4 m	6	5 d/wk	Zn + Fe supplement	Sulfate	10	7.1	↓

Fahmida [26]	2007	Indonesia	303	Anemic infants (80% anemia)	3-6 m	6	Daily	Zn supplement	Sulfate	10	10	±
Heinig [8]	2006	USA	19	Healthy infants	4 m	6	Daily	Zn supplement	Gluconate or Sulfate	5	5	±
Hess [35]	2015	Burkina Faso	970	Healthy infants	9 m	9	Daily	Zn supplement	Not specified, but likely sulfate	5	5	±
Hess [35]	2015	Burkina Faso	987	Healthy infants	9 m	9	Daily	Zn + Fe SQ-LNS + placebo supplement	Not specified, but likely sulfate	5	5	±
Hess [35]	2015	Burkina Faso	978	Healthy infants	9 m	9	Daily	Zn + Fe SQ-LNS + placebo supplement	Not specified, but likely sulfate	10	10	±
Lind [9]	2003	Indonesia	277	Healthy infants	6 m	6	Daily	Zn supplement	Sulfate	10	10	±
Lind [9]	2003	Indonesia	272	Healthy infants	6 m	6	Daily	Zn + Fe supplement	Sulfate	10	10	↓
Lopez de Romaña [27]	2005	Peru	22	Stunted and anemic children	3-4 y	1.6	5d/wk	Zn + Fe fortified wheat	Sulfate	3	2.1	±
Lopez de Romaña [27]	2005	Peru	24	Stunted and anemic children	3-4 y	1.6	5d/wk	Zn + Fe fortified wheat	Sulfate	9	6.4	↓ [±]*
Olney [36]	2006	Zanzibar	102	Nutritionally at-risk infants	9.0 m	12	Daily	Zn supplement	Not specified, but likely sulfate	10	10	↑
Olney [36]	2006	Zanzibar	110	Nutritionally at-risk infants	8.6-8.7 m	12	Daily	Zn + Fe + FA supplement	Not specified, but likely sulfate	10	10	±
Owusu-Agyei [37]	2013	Ghana	182	Healthy infants and children	6-24 m	6	Daily	Zn supplement (+ 1 dose VitA)	Gluconate	10	10	[±]
Penny [38]	2004	Peru	134	Infants and children recovering from persistent diarrhea	6-36 m	6	Daily	Zn supplement	Gluconate	10	10	±

Radhakrishna [10]	2013	India	68	Healthy infants	4 m	14 (12 with suppl)	Daily	Zn+B2 supplement	Sulfate	5	5	±
Richard [39]	2006	Peru	418	Healthy infants and children	6 m to 15 y	7	6d/wk	Zn supplement	Sulfate	20	17.1	±
Richard [39]	2006	Peru	418	Healthy infants and children	6 m to 15 y	7	6d/wk	Fe + Zn supplement	Sulfate	20	17.1	±
Rosado [11]	2006	Mexico	187	Healthy children with environmental lead exposure	6-7 y	6	5d/wk	Zn supplement	Oxide	30	21.4	±
Rosado [11]	2006	Mexico	198	Healthy children with environmental lead exposure	6-7 y	6	5d/wk	Fe + Zn supplement	Oxide	30	21.4	±
Rosado [28]	1997	Mexico	95	Healthy children	18-36 m	12	5d/wk	Zn supplement	Methionine	20	14.3	±
Rosado [28]	1997	Mexico	99	Healthy children	18-36 m	12	5d/wk	Fe + Zn supplement	Methionine	20	14.3	±
Ruz [12]	1997	Chile	74	Healthy children	27-50 m	6 biochem	Daily	Intervention; Zn supplement	Sulfate	10	10	±
Sazawal [13]	2004	India	115	Healthy infants	5-12 m	4	Daily	Zn supplement + multi-vitamins (no zinc)	Gluconate	10	10	±
Sazawal [40]	2018	India	6005	Healthy children	4-6 y	6	Daily	High Zn biofortified wheat (control is low zinc biofortified wheat)	Not specified	1.2 diff from lo zinc control	»1.2	±
Shankar [41]	2000	Papua New Guinea	212	Infants and children in malaria endemic area	6-60 m	10.6	6d/wk	Zn supplement	Gluconate	10	8.6	±
Smith [42]	1999	El Salvador	37	Children with low serum Zn and low VitA	28-70 m	6	Weekly	Zn supplement	Gluconate	Intervention:70	10	±
Smith [42]	1999	El Salvador	37	Children with low serum Zn and low VitA	28-70 m	6	Weekly	Zn + VitA supplement	Gluconate	Intervention:70	10	±
Soofi [29]	2013	Pakistan	411	Healthy infants	6 m	12	Daily	Zn + Fe + MNP supplement	Gluconate	10	10	±

Taneja [43]	2009	India	319	Healthy infants	0.5 m	9	Daily	Zn + Fe + MN supplement	Sulfate	5 for 3-5 m-old, 10 for 6-12 m-old	5 for 3-5 m-old, 10 for 6-12 m-old	±
Tielsch [30]	2006	Nepal	335	Healthy infants and children	1-35 m	12	Daily	Zn + Fe + FA supplement	Sulfate	5 for 1-11 m-old, 10 for 12-35 m-old	5 for 1-11 m-old, 10 for 12-35 m-old	±
Veenemans [31]	2011	Tanzania	299	Healthy infants and children	6-60 m	8.3	Daily	Zn supplement	Gluconate	10	10	±
Veenemans [31]	2011	Tanzania	299	Healthy infants and children	6-60 m	8.3	Daily	Zn + MMN with Fe supplement	Gluconate	10	10	±
Wasantwisut [32]	2006	Thailand	124	Healthy infants, Hb 80+ g/L	4-6 m	6	Daily	Zn + VitC supplement	Sulfate	10	10	↓ [±]
Wasantwisut [32]	2006	Thailand	132	Healthy infants, Hb 80+ g/L	4-6 m	6	Daily	Zn + Fe + VitC supplement	Sulfate	10	10	↓ [±]
Wieringa [33]	2003	Indonesia	91	Healthy infants	4 m	6	5d/wk	Zn supplement	Sulfate	10	7.1	±
Wieringa [33]	2003	Indonesia	77	Healthy infants	4 m	6	5d/wk	Zn + β-carotene supplement	Sulfate	10	7.1	±
Wieringa [33]	2003	Indonesia	88	Healthy infants	4 m	6	5d/wk	Zn + Fe supplement	Sulfate	10	7.1	±
Wuehler [17]	2008	Ecuador	106	Healthy children	12-30 m	6	Daily	Zn supplement	Sulfate	3	3	±
Wuehler [17]	2008	Ecuador	108	Healthy children	12-30 m	6	Daily	Zn supplement	Sulfate	7	7	±
Wuehler [17]	2008	Ecuador	110	Healthy children	12-30 m	6	Daily	Zn supplement	Sulfate	10	10	±
Zlotkin [34]	2003	Ghana	239	Infants and children with mild-moderate anemia	6-18 m	2	Daily	Zn + Fe + VitC supplement	Gluconate	10	10	↓

*In brackets is the effect as reported by the original study. ↑ or ↓ indicates the final concentration is higher or lower, respectively, in the zinc intervention group compared to the non-zinc comparison; ± indicates no reported difference between zinc and no-zinc interventions.

[1] All control groups received the same as the intervention except the dose of zinc listed

Table S6. Studies and comparisons with reported serum transferrin receptor outcomes in response to additional zinc supplementation or fortification, by author.

Study	Year	Country	Sample size analyzed	Characteristics of subjects	Initial age	Duration, m	Schedule	Intervention (All controls are same without the added zinc)	Form of zinc	Dose[1], mg	Actual dose[1], mg/d	Effect of zinc§
Baqui [1]	2005	Bangladesh	80	Healthy infants	6 m	6	Weekly	Zn + B_2 supplement	Acetate	20	2.9	±
Baqui [1]	2005	Bangladesh	83	Healthy infants	6 m	6	Weekly	Zn + Fe + B_2 supplement	Acetate	20	2.9	±
Becquey [22] data from authors	2016	Burkina Faso	165	Healthy infants and children	6-27 m	11	Daily	Zn supplement	Sulfate	7	7	±
Becquey [22] data from authors	2016	Burkina Faso	169	Healthy infants and children	6-27 m	11	10 d every 16 wk	Zn supplement	Sulfate	1.4	0.9	±
Carter [24]	2018	Tanzania	302	Healthy infants	5-7 wk	6 (18)	Daily	Zn supplement	Sulfate	5 until 6 m of age then 10	5 until 6 m of age then 10	±
Carter [24]	2018	Tanzania	291	Healthy infants	5-7 wk	18	Daily	Zn + MV (no Fe) supplement	Sulfate	5 until 6 m of age then 10	5 until 6 m of age then 10	±
Lind [9]	2003	Indonesia	257	Healthy infants	6 m	6	Daily	Zn supplement	Sulfate	10	10	±
Lind [9]	2003	Indonesia	250	Healthy infants	6 m	6	Daily	Zn + Fe supplement	Sulfate	10	10	±

*In brackets is the effect as reported by the original study. ↑ or ↓ indicates the final concentration is higher or lower, respectively, in the zinc intervention group compared to the non-zinc comparison; ± indicates no reported difference between zinc and no-zinc interventions.

[1] All control groups received the same as the intervention except the dose of zinc listed

Table S7. Studies and comparisons with reported lipid outcomes in response to additional zinc supplementation or fortification, by author.

**Study**	**Year**	**Country**	**Sample size analyzed**	**Characteristics of subjects**	**Initial age**	**Duration, m**	**Schedule**	**Intervention (All controls are same without the added zinc)**	**Form of zinc**	**Dose¹, mg**	**Actual dose¹, mg/d**	**Effect of zinc§**
Chimhashu [44]	2018	Benin	162	Healthy children	6-10 y	4.6	5 d/wk	Zn fortified water	Zinc ions	4.3	2.8	nervonic acid
Hambidge [7]	1979	USA	58	Healthy children	33-90 m	9	Daily	Zn fortified breakfast cereal (control, no fortificant)	Oxide	9.3	3	± serum cholesterol
Walravens [15]	1976	USA	68	Healthy infants	4-6 d	6	Daily	Zn + Fe fortified formula	Sulfate	5.8	4	± serum cholesterol
Wuehler [17]	2008	Ecuador	110	Healthy children	12-30 m	6	Daily	Zn supplement	Sulfate	10	10	± serum cholesterol

*In brackets is the effect as reported by the original study. ↑ or ↓ indicates the final concentration is higher or lower, respectively, in the zinc intervention group compared to the non-zinc comparison; ± indicates no reported difference between zinc and no-zinc interventions.

¹ All control groups received the same as the intervention except the dose of zinc listed

Supplementary Figures

Figure S1: Effect of additional zinc intervention on plasma or serum ceruloplasmin concentrations.

	zinc			no zinc				Std. Mean Difference
**Study or Subgroup**	**Mean**	**SD**	**Total**	**Mean**	**SD**	**Total**	**Weight**	**IV, Random, 95% CI**
05.0mg/d_Bertinato 2013 (Zn-s vs P-s)	6	8	10	12	7	3	19.1%	-0.71 [-2.04, 0.62]
10mg/d_Bertinato 2013 (Zn-s vs P-s)	4	8	9	12	7	3	18.8%	-0.95 [-2.33, 0.44]
10mg/d_De Brito 2014 (Zn-s vs P-s)	276	11.7	15	324	15.3	15	20.0%	-3.43 [-4.60, -2.25]
10mg/d_Wuehler 2008 (Zn-s vs P-s)	-18.3	62	53	-23.4	55.2	49	23.3%	0.09 [-0.30, 0.47]
15mg/d_Bertinato 2013 (Zn-s vs P-s)	6	8	8	12	7	3	18.9%	-0.70 [-2.08, 0.67]
**Total (95% CI)**			**95**			**73**	**100.0%**	**-1.11 [-2.43, 0.20]**

Heterogeneity: Tau² = 1.90; Chi² = 32.50, df = 4 (P < 0.00001); I² = 88%
Test for overall effect: Z = 1.66 (P = 0.10)

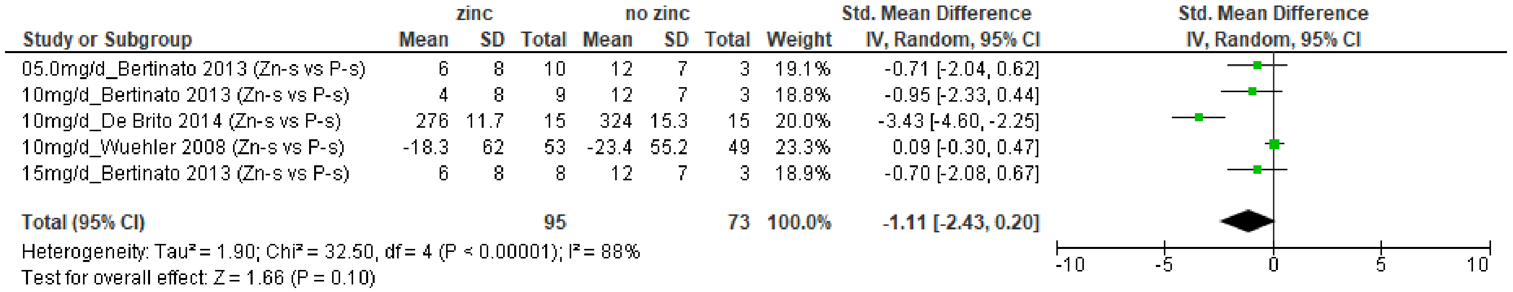

Figure S1 legends: *Zn-s: Zinc supplement; P-s: Placebo supplement*

Figure S2: Effect of additional zinc intervention on erythrocyte super-oxide dismutase concentrations.

Study or Subgroup	Mean	SD	Total	Mean	SD	Total	Weight	Std. Mean Difference IV, Random, 95% CI
05.0mg/d_Bertinato 2013 (Zn-s vs P-s)	-50	440	10	-150	440	3	6.0%	0.21 [-1.08, 1.51]
05.0mg/d_Heinig 2006 (Zn-s vs P-s)	1,080	660	7	1,430	1,160	12	10.6%	-0.33 [-1.27, 0.61]
10mg/d_Bertinato 2013 (Zn-s vs P-s)	-740	460	9	-150	440	3	5.0%	-1.19 [-2.62, 0.24]
10mg/d_Wuehler 2008 (Zn-s vs P-s)	2.1	7.1	53	-0.4	5.5	49	35.9%	0.39 [-0.00, 0.78]
15mg/d_Bertinato 2013 (Zn-s vs P-s)	100	490	8	-150	440	3	5.6%	0.48 [-0.87, 1.83]
20mg/d_Bates 1993 (Zn-s vs P-s)	3.3	582.4	53	3.3	660	53	36.9%	0.00 [-0.38, 0.38]
**Total (95% CI)**			**140**			**123**	**100.0%**	**0.08 [-0.25, 0.42]**

Heterogeneity: Tau² = 0.04; Chi² = 6.60, df = 5 (P = 0.25); I² = 24%
Test for overall effect: Z = 0.50 (P = 0.62)

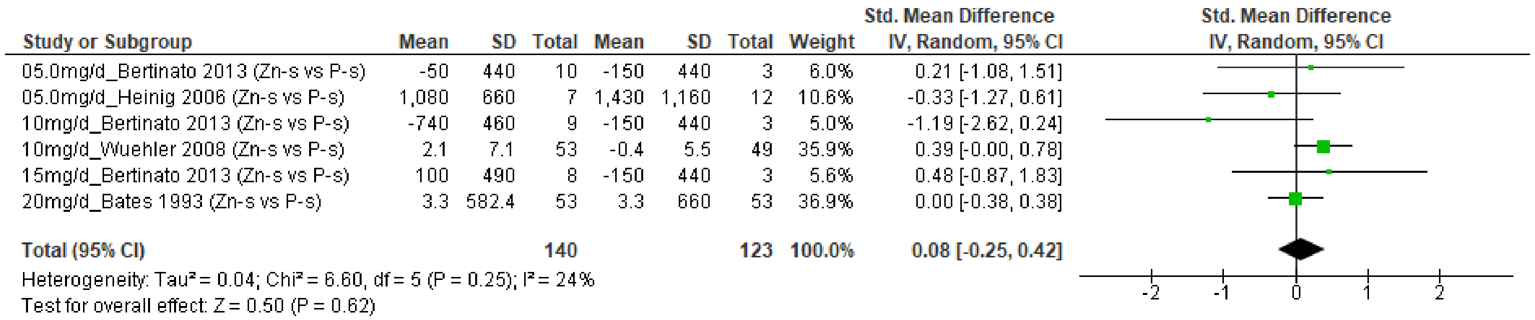

Figure S2 legends: *Zn-s: Zinc supplement; P-s: Placebo supplement*

Figure S3: Effect of additional zinc intervention (no iron was provided either in the intervention or control group) on serum ferritin concentrations (a) < 12 months, (b) 12 months or more.

Study or Subgroup	zinc Mean	zinc SD	zinc Total	no zinc Mean	no zinc SD	no zinc Total	Weight	Std. Mean Difference IV, Random, 95% CI
**(a) < 12 months**								
02.9mg/d_Baqui 2005 (Zn+B2-s vs B2-s)	-15.3	22.6	40	-21.7	27.7	37	4.0%	0.25 [-0.20, 0.70]
05.0mg/d_Heinig 2006 (Zn-s vs P-s)	35.7	22	7	41.8	14.7	12	1.4%	-0.33 [-1.27, 0.61]
07.1mg/d_Dijkhuizen 2001 (Zn-s vs P-s)	13.1	11.3	97	15.9	12.7	87	5.9%	-0.23 [-0.52, 0.06]
07.1mg/d_Wieringa 2003 (Zn+BCaro-s vs BCaro-s)	12.4	12.2	39	12.3	12.4	38	4.1%	0.01 [-0.44, 0.45]
07.1mg/d_Wieringa 2003 (Zn-s vs P-s)	11.6	9.7	48	17.2	14.5	43	4.4%	-0.46 [-0.87, -0.04]
10mg/d_Berger 2006 (Zn-s vs P-s)	16.5	12.4	173	16.5	12.5	176	7.0%	0.00 [-0.21, 0.21]
10mg/d_Carter 2018 (Zn+MV-s vs MV-s)	51.7	46.1	144	51.3	46.1	147	6.8%	0.01 [-0.22, 0.24]
10mg/d_Carter 2018 (Zn-s vs P-s)	50.7	46.1	151	48.1	46.1	151	6.8%	0.06 [-0.17, 0.28]
10mg/d_Fahmida 2007 (Zn-s vs P-s)	9.4	3.5	25	14.1	2.7	34	2.9%	-1.51 [-2.10, -0.93]
10mg/d_Lind 2003 (Zn-s vs P-s)	13.3	3.6	134	13.9	3.5	72	6.0%	-0.17 [-0.45, 0.12]
10mg/d_Wasantwisut 2006 (Zn+VC-s vs VC-s)	28.5	22.2	58	26.7	13.8	66	5.1%	0.10 [-0.25, 0.45]
**Subtotal (95% CI)**			**916**			**863**	**54.4%**	**-0.14 [-0.33, 0.04]**
Heterogeneity: Tau² = 0.06; Chi² = 33.36, df = 10 (P = 0.0002); I² = 70%								
Test for overall effect: Z = 1.51 (P = 0.13)								
**(b) 12 months and older**								
0.9mg/d_Becquey 2016 (Zn-s vs no-s)	11.7	11.9	118	14.9	11.3	127	6.5%	-0.28 [-0.53, -0.02]
07.0mg/d_Becquey 2016 (Zn-s vs no-s)	12.1	12.2	123	14.9	11.3	127	6.5%	-0.24 [-0.49, 0.01]
10mg/d_De Brito 2014 (Zn-s vs P-s)	34	3.5	15	34.2	3.3	15	2.2%	-0.06 [-0.77, 0.66]
10mg/d_Ruz 1997 (Zn-s vs P-s)	23.3	11	49	26.3	13.8	49	4.6%	-0.24 [-0.64, 0.16]
10mg/d_Veenemans 2011 (Zn-s vs P-s)	23.2	2.3	95	24.4	2.4	97	6.0%	-0.51 [-0.80, -0.22]
10mg/d_Wuehler 2008 (Zn-s vs P-s)	21.5	5.6	54	19.9	4.7	56	4.8%	0.31 [-0.07, 0.68]
14.3mg/d_Rosado 1997 (Zn-s vs P-s)	19.7	13.5	48	22.9	16.5	48	4.5%	-0.21 [-0.61, 0.19]
20mg/d_Bates 1993 (Zn-s vs P-s)	17.2	16	40	15.9	17.6	45	4.3%	0.08 [-0.35, 0.50]
21.4mg/d_Rosado 2006 (Zn-s vs P-s)	14.1	28.8	105	16.2	28.8	115	6.3%	-0.07 [-0.34, 0.19]
**Subtotal (95% CI)**			**647**			**679**	**45.6%**	**-0.16 [-0.32, -0.01]**
Heterogeneity: Tau² = 0.02; Chi² = 14.46, df = 8 (P = 0.07); I² = 45%								
Test for overall effect: Z = 2.12 (P = 0.03)								
**Total (95% CI)**			**1563**			**1542**	**100.0%**	**-0.14 [-0.27, -0.02]**
Heterogeneity: Tau² = 0.04; Chi² = 49.92, df = 19 (P = 0.0001); I² = 62%								
Test for overall effect: Z = 2.31 (P = 0.02)								
Test for subgroup differences: Chi² = 0.03, df = 1 (P = 0.86), I² = 0%								

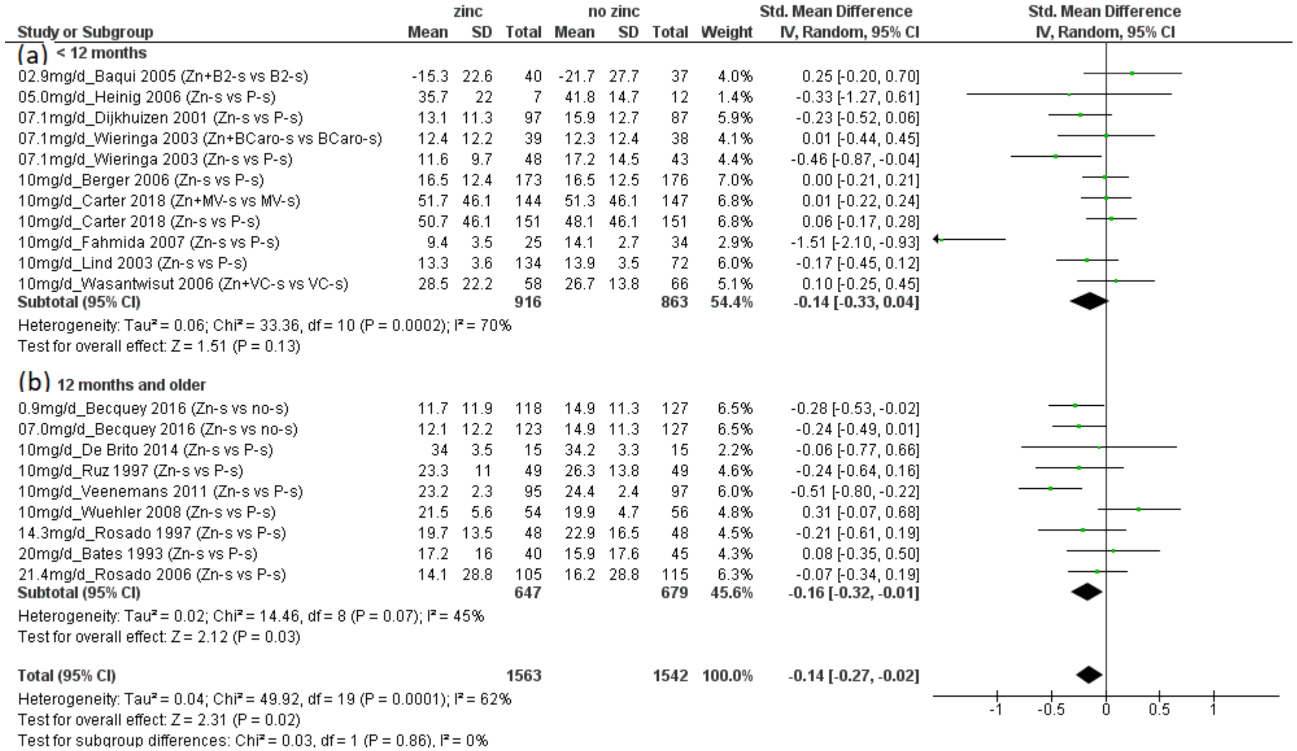

Figure S3 legends: Zn-s: Zinc supplement; Zn + B2-s: Zinc plus vitamin B12 supplement; B2-s: Vitamin B2 supplement; P-s: Placebo supplement; no-s: No supplement; Zn + BCaro-s: zinc plus beta carotene supplement; BCaro-s: Beta carotene supplement; Zn + MV-s: Zinc plus multivitamin supplement; MV-s: Multivitamin supplement; Zn + VC-s: Zinc plus vitamin C supplement; VC-s: Vitamin C supplement. NOTE: Placebo value (P) from Lind et al 2003 updated by Dr. Lind through personal communication.

Figure S4: Effect of additional zinc intervention (iron was provided in both the intervention and control group) on serum ferritin concentrations (a) < 12 months, (b) 12 months or more.

Study or Subgroup	zinc Mean	zinc SD	zinc Total	no zinc Mean	no zinc SD	no zinc Total	Weight	Std. Mean Difference IV, Random, 95% CI
**(a) < 12 months**								
02.3mg/d_Brown 2007 (Zn-s+Fe-f vs P-s+Fe-f)	-20.9	28.7	74	-14.3	27.4	42	5.3%	-0.23 [-0.61, 0.15]
02.9mg/d_Baqui 2005 (Zn+Fe+B2-s vs Fe+B2-s)	-13.7	24.7	41	-18	26.8	38	5.3%	0.17 [-0.28, 0.61]
03.3 mg/d_Brown 2007 (Zn+Fe-f vs Fe-f)	-15.3	43.4	76	-14.3	27.4	42	5.3%	-0.03 [-0.40, 0.35]
07.1mg/d_Dijkhuizen 2001 (Zn+Fe-s vs Fe-s)	28.4	18.4	74	39	31.2	90	5.4%	-0.40 [-0.71, -0.09]
07.1mg/d_Wieringa 2003 (Zn+Fe-s vs Fe-s)	30.2	17.9	39	41.4	37.1	49	5.3%	-0.37 [-0.79, 0.06]
10mg/d_Berger 2006 (Zn+Fe-s vs Fe-s)	52.3	42.9	190	58	48.1	201	5.5%	-0.12 [-0.32, 0.07]
10mg/d_Caulfield 2013 (Zn+Fe+Cu-s vs Fe+Cu-s)	9	12.1	85	11.8	17.4	85	5.4%	-0.19 [-0.49, 0.12]
10mg/d_Lind 2003 (Zn+Fe-s vs Fe-s)	32.3	2.9	136	46.5	2	143	5.1%	-5.71 [-6.24, -5.18]
10mg/d_Soofi 2013 (Zn in MNP vs MNP with no Zn)	16	16.7	158	19.1	17.7	159	5.5%	-0.18 [-0.40, 0.04]
10mg/d_Wasantwisut 2006 (Zn+Fe+VC-s vs Fe+VC-s)	60.1	50.2	67	77.5	23.5	65	5.4%	-0.44 [-0.78, -0.09]
10mg/d_Zlotkin 2003 (Zn+Fe+VC-s vs Fe+VC-s)	51	96	110	37.4	97.2	92	5.4%	0.14 [-0.14, 0.42]
**Subtotal (95% CI)**			**1050**			**1006**	**58.9%**	**-0.65 [-1.26, -0.04]**
Heterogeneity: Tau² = 1.04; Chi² = 419.25, df = 10 (P < 0.00001); I² = 98%								
Test for overall effect: Z = 2.08 (P = 0.04)								
**(b) 12 months and older**								
02.1mg/d_Lopez de Romana 2005 (Zn+Fe-f vs Fe-f)	12.2	13	12	15.2	20.3	6	4.4%	-0.18 [-1.16, 0.80]
05.0mg_Abdollahi 2014 (Zn+Fe-s vs Fe-s)	47.7	43.1	272	45.6	41	308	5.5%	0.05 [-0.11, 0.21]
06.4mg/d_Lopez de Romana 2005 (Zn+Fe-f vs Fe-f)	9.6	14	12	15.2	20.3	6	4.4%	-0.33 [-1.32, 0.66]
06.5mg/d_Alarcon 2004 (Zn+Fe-s vs Fe-s)	37.4	14.6	109	28	9.6	104	5.4%	0.75 [0.48, 1.03]
10mg/d_Tielsch 2006 (Zn+Fe+FA-s vs Fe+FA-s)	35.9	23	164	53.6	34.9	146	5.5%	-0.60 [-0.83, -0.38]
10mg/d_Veenemans 2011 (Zn+MN-s vs MN-s)	43.8	1.77	98	51.1	2	113	5.2%	-3.83 [-4.29, -3.38]
14.3mg/d_Rosado 1997 (Zn+Fe-s vs Fe-s)	43.6	22.9	48	46.9	24.2	48	5.3%	-0.14 [-0.54, 0.26]
21.4 mg/d_Rosado 2006 (Zn+Fe-s vs Fe-s)	41.4	28.8	113	53.7	28.8	119	5.4%	-0.43 [-0.69, -0.17]
**Subtotal (95% CI)**			**828**			**850**	**41.1%**	**-0.59 [-1.33, 0.15]**
Heterogeneity: Tau² = 1.06; Chi² = 309.85, df = 7 (P < 0.00001); I² = 98%								
Test for overall effect: Z = 1.57 (P = 0.12)								
**Total (95% CI)**			**1878**			**1856**	**100.0%**	**-0.63 [-1.07, -0.18]**
Heterogeneity: Tau² = 0.95; Chi² = 729.84, df = 18 (P < 0.00001); I² = 98%								
Test for overall effect: Z = 2.73 (P = 0.006)								
Test for subgroup differences: Chi² = 0.02, df = 1 (P = 0.90), I² = 0%								

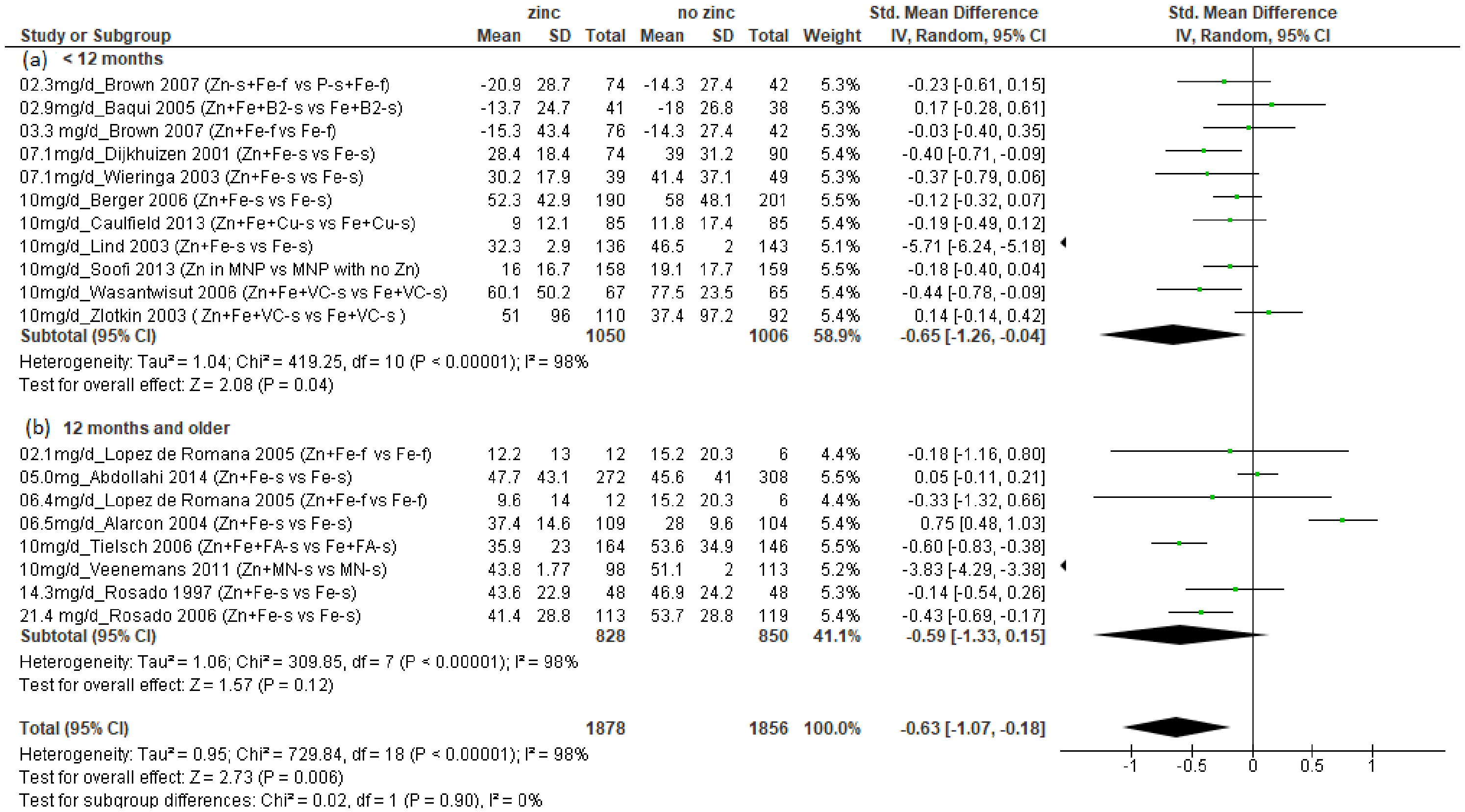

Figure S4 legends: Zn+Fe-s: Zinc plus iron supplement; Zn + Fe + Cu-s: Zinc plus iron plus copper supplement; Fe + Cu-s: Iron plus copper supplement; Zn+Fe+B2-s: Zinc plus iron plus vitamin B2 supplement; Fe-f: Iron fortified; Fe-s: Iron supplement; Zn + Fe-f: Zinc plus iron fortified; Fe-f: Iron fortified; Zn-s+Fe-f: Zinc supplement plus iron fortified; P-s+Fe-f: Placebo supplement plus iron fortified; Zn+Fe+FA-s: Zinc plus iron plus folic acid supplement; Fe+FA-s: Iron plus folic acid supplement; Zn in MNP: Micronutrient powder with zinc plus iron; MNP with no Zn: Micronutrient powder with no zinc plus iron; Zn + MN: Zinc plus micronutrient supplement; Zn + Fe + VC-s: Zinc plus iron plus vitamin C supplement.

Figure S5: Effect of additional zinc intervention on whole blood hemoglobin concentrations, by mean age at baseline: (a) <12 months, (b) 12 months or older.

	zinc			no zinc				Std. Mean Difference
**Study or Subgroup**	**Mean**	**SD**	**Total**	**Mean**	**SD**	**Total**	**Weight**	**IV, Random, 95% CI**
**(a) < 12 months**								
02.3mg/d_Brown 2007 (Zn-s+Fe-f vs P-s+Fe-f)	-0.1	12.6	74	-2.6	10.5	42	1.4%	0.21 [-0.17, 0.59]
02.9mg/d_Baqui 2005 (Zn+B2-s vs B2-s)	-3.9	12.8	64	-1.61	13.9	60	1.5%	-0.17 [-0.52, 0.18]
02.9mg/d_Baqui 2005 (Zn+Fe+B2-s vs Fe+B2-s)	-1.8	12.8	57	1.6	12.8	68	1.5%	-0.26 [-0.62, 0.09]
03.3 mg/d_Brown 2007 (Zn+Fe-f vs Fe-f)	-1.2	10.8	76	-2.6	10.5	42	1.4%	0.13 [-0.25, 0.51]
05.0mg/d_Heinig 2006 (Zn-s vs P-s)	129	16	7	130.8	8.9	12	0.3%	-0.14 [-1.08, 0.79]
05.0mg/d_Hess 2015 (SQ-LNS:Zn vs SQ-LNS:No Zn)	90	16	613	89	15	200	3.0%	0.06 [-0.10, 0.22]
05.0mg/d_Hess 2015 (Zn-s vs P-s)	90	15	617	89	15	200	3.0%	0.07 [-0.09, 0.23]
05.0mg/d_Radhakrishna 2013 (Zn-s vs P-s)	84	14.1	34	86.3	15.6	34	1.0%	-0.15 [-0.63, 0.32]
07.1mg/d_Dijkhuizen 2001 (Zn+Fe-s vs Fe-s)	110	11	74	115	11	90	1.8%	-0.45 [-0.76, -0.14]
07.1mg/d_Dijkhuizen 2001 (Zn-s vs P-s)	106	11	97	106	11	87	1.9%	0.00 [-0.29, 0.29]
07.1mg/d_Wieringa 2003 (Zn+Bcaro-s vs Bcaro-s)	107	10	39	112	9	38	1.1%	-0.52 [-0.97, -0.07]
07.1mg/d_Wieringa 2003 (Zn+Fe-s vs Fe-s)	115	9	39	118	10	49	1.2%	-0.31 [-0.73, 0.11]
07.1mg/d_Wieringa 2003 (Zn-s vs P-s)	110	10	48	110	11	43	1.3%	0.00 [-0.41, 0.41]
10mg/d_Berger 2006(Zn+Fe-s vs Fe-s)	129.3	15.9	190	131.8	16.7	201	2.6%	-0.15 [-0.35, 0.05]
10mg/d_Berger 2006 (Zn-s vs P-s)	117.8	16.1	191	118.3	15.7	195	2.6%	-0.03 [-0.23, 0.17]
10mg/d_Brooks 2005 (Zn-s vs P-s)	96	9.1	329	97	9.4	309	3.0%	-0.11 [-0.26, 0.05]
10mg/d_Carter 2018 (Zn+MV-s vs MV-s)	99	19.8	167	99	13.1	165	2.5%	0.00 [-0.22, 0.22]
10mg/d_Carter 2018 (Zn-s vs P-s)	96	9.7	159	96	13.2	168	2.5%	0.00 [-0.22, 0.22]
10mg/d_Caulfield 2013 (Zn+Fe+Cu-s vs Fe+Cu-s)	117.3	8.7	101	115.2	9.1	108	2.0%	0.23 [-0.04, 0.51]
10mg/d_Fahmida 2007 (Zn-s vs P-s)	9	1.5	25	9.1	1.5	34	0.9%	-0.07 [-0.58, 0.45]
10mg/d_Hess 2015 (SQ-LNS:Zn vs SQ-LNS:no Zn)	89	15	603	89	15	200	3.0%	0.00 [-0.16, 0.16]
10mg/d_Lind 2003 (Zn+Fe-s vs Fe-s)	115.3	13.9	136	119.4	15.3	143	2.3%	-0.28 [-0.52, -0.04]
10mg/d_Lind 2003 (Zn-s vs P-s)	115.7	15.2	134	113.5	16	143	2.3%	0.14 [-0.10, 0.38]
10mg/d_Olney 2006 (Zn+Fe+FA-s vs Fe+FA-s)	1.3	10.9	56	4.2	11.9	54	1.4%	-0.25 [-0.63, 0.12]
10mg/d_Olney 2006 (Zn-s vs P-s)	3.4	11.5	44	-7.5	11.4	58	1.3%	0.95 [0.53, 1.36]
10mg/d_Sazawal 2004 (Zn-s vs P-s)	101	20	58	101	8	52	1.4%	0.00 [-0.37, 0.37]
10mg/d_Soofi 2013 (Zn in MNP+Fe vs MNP with no Zn)	101	14.8	210	102	15	217	2.7%	-0.07 [-0.26, 0.12]
10mg/d_Taneja 2009 (Zn+MN-s vs MN-s)	94	14	151	96	14	168	2.4%	-0.14 [-0.36, 0.08]
10mg/d_Wasantwisut 2006 (Zn+Fe+VC-s vs Fe+VC-s)	112.9	11.4	67	118	12.2	65	1.6%	-0.43 [-0.77, -0.08]
10mg/d_Wasantwisut 2006 (Zn+VC-s vs VC-s)	101.6	11.4	58	106.3	13	66	1.5%	-0.38 [-0.74, -0.02]
10mg/d_Zlotkin 2003 (Zn+Fe+VC-s vs Fe+VC-s)	103.5	15.8	124	108.1	15.5	115	2.2%	-0.29 [-0.55, -0.04]
**Subtotal (95% CI)**			**4642**			**3426**	**58.9%**	**-0.07 [-0.14, 0.01]**
Heterogeneity: Tau^2 = 0.02; Chi^2 = 69.13, df = 30 (P < 0.0001); I^2 = 57%								
Test for overall effect: Z = 1.83 (P = 0.07)								
**(b) 12 months or older**								
0.9mg/d_Becquey 2016 (Zn-s vs no-s)	100.7	20.6	123	98.9	20.3	127	2.2%	0.09 [-0.16, 0.34]
01.2mg/d_Sazawal 2018 (Zn-H-f vs Zn-L-f)	114	15.7	2997	114.6	16.1	3008	3.8%	-0.04 [-0.09, 0.01]
02.1mg/d_Lopez de Romana 2005 (Zn+Fe-f vs Fe-f)	124	17.3	10	129.6	13.9	6	0.3%	-0.33 [-1.35, 0.69]
03.0mg/d_Wuehler 2008 (Zn-s vs P-s)	-3.1	13.7	50	-6.8	14.1	18	0.9%	0.26 [-0.28, 0.81]
06.4mg/d_Lopez de Romana 2005 (Zn+Fe-f vs Fe-f)	117.4	10.1	12	129.6	13.9	6	0.3%	-1.02 [-2.07, 0.03]
06.5mg/d_Alarcon 2004 (Zn+Fe-s vs Fe-s)	119.1	7.1	109	114.7	6.2	104	2.0%	0.66 [0.38, 0.93]
07.0mg/d_Becquey 2016 (Zn-s vs no-s)	97.5	21.1	123	98.9	20.3	127	2.2%	-0.07 [-0.32, 0.18]
07.0mg/d_Wuehler 2008 (Zn-s vs P-s)	-4.3	15.1	52	-6.8	14.1	19	0.9%	0.17 [-0.36, 0.69]
08.6mg/d_Shankar 2000 (Zn-s vs P-s)	86	13	103	87	17	109	2.1%	-0.07 [-0.34, 0.20]
10mg/d_De Brito 2014 (Zn-s vs P-s)	116	1.7	15	117	2	15	0.5%	-0.52 [-1.25, 0.21]
10mg/d_Owusu-Agyei 2013 (Zn+VA-s vs VA-s)	109	1.4	100	108	1.4	100	2.0%	0.71 [0.43, 1.00]
10mg/d_Penny 2004 (Zn-s vs P-s)	3.3	14.1	69	8.3	13.2	69	1.6%	-0.36 [-0.70, -0.03]
10mg/d_Ruz 1997 (Zn-s vs P-s)	125	89	40	124	85	34	1.1%	0.01 [-0.45, 0.47]
10mg/d_Smith 1999 (Zn+VA-s vs VA-s)	116	7.1	7	117	7.1	10	0.3%	-0.13 [-1.10, 0.83]
10mg/d_Smith 1999 (Zn-s vs P-s)	113	8.4	11	109	8.4	9	0.4%	0.46 [-0.44, 1.35]
10mg/d_Tielsch 2006 (Zn+Fe+FA-s vs Fe+FA-s)	109.6	16.1	182	111.1	11.8	152	2.5%	-0.10 [-0.32, 0.11]
10mg/d_Veenemans 2011 (Zn+MN-s vs MN-s)	107.5	11.4	148	106.6	10.7	151	2.4%	0.08 [-0.15, 0.31]
10mg/d_Veenemans 2011 (Zn-s vs P-s)	103.7	11.8	149	104	11.9	150	2.4%	-0.03 [-0.25, 0.20]
10mg/d_Wuehler 2008 (Zn-s vs P-s)	-3.9	13.3	54	-6.8	14.1	19	0.9%	0.21 [-0.31, 0.74]
14.3mg/d_Rosado 1997 (Zn+Fe-s vs Fe-s)	118	7	48	118	9	48	1.3%	0.00 [-0.40, 0.40]
14.3mg/d_Rosado 1997 (Zn-s vs P-s)	118	7	48	118	8	48	1.3%	0.00 [-0.40, 0.40]
17.1mg/d_Richard 2006 (Zn+Fe-s vs Fe-s)	119.1	10.5	214	119	10.9	212	2.7%	0.01 [-0.18, 0.20]
17.1mg/d_Richard 2006 (Zn-s vs P-s)	116.6	11.9	214	116.9	12.4	215	2.7%	-0.02 [-0.21, 0.16]
21.4 mg/d_Rosado 2006 (Zn+Fe-s vs Fe-s)	1.2	6.2	118	1.8	7.2	133	2.2%	-0.09 [-0.34, 0.16]
21.4mg/d_Rosado 2006 (Zn-s vs P-s)	1.2	6.4	113	0.5	7	123	2.2%	0.10 [-0.15, 0.36]
**Subtotal (95% CI)**			**5109**			**5012**	**41.1%**	**0.04 [-0.05, 0.14]**
Heterogeneity: Tau^2 = 0.03; Chi^2 = 64.78, df = 24 (P < 0.0001); I^2 = 63%								
Test for overall effect: Z = 0.93 (P = 0.35)								
**Total (95% CI)**			**9751**			**8438**	**100.0%**	**-0.02 [-0.08, 0.03]**
Heterogeneity: Tau^2 = 0.02; Chi^2 = 136.83, df = 55 (P < 0.00001); I^2 = 60%								
Test for overall effect: Z = 0.79 (P = 0.43)								
Test for subgroup differences: Chi^2 = 3.49, df = 1 (P = 0.06), I^2 = 71.4%								

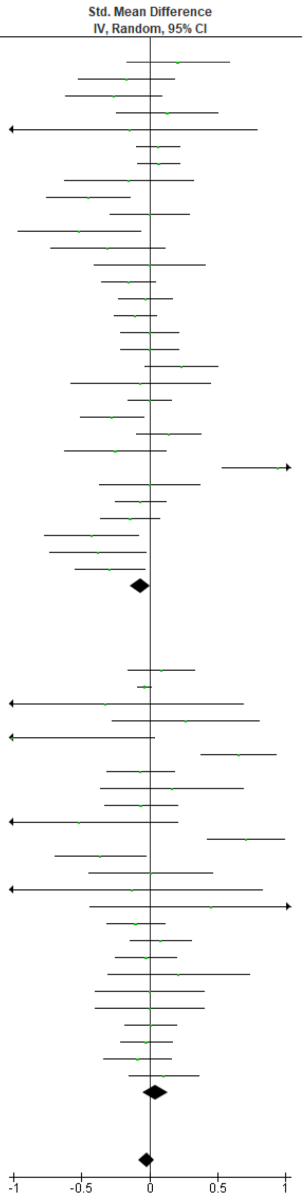

Figure S5 legends: Zn-s: Zinc supplement; Zn+ Fe-s: Zinc plus iron supplement; Zn + Fe + Cu-s: Zinc plus iron plus copper supplement; Fe + Cu-s: Iron plus copper supplement; Zn + B2-s: Zinc plus vitamin B12 supplement; B2-s: Vitamin B2 supplement; Zn+Fe+B2-s: Zinc plus iron plus vitamin B2 supplement; Zn-f: Zinc fortified; P-s: Placebo supplement; P-f: Placebo fortified; Fe-f: Iron fortified; Fe-s: Iron supplement; Zn-H-Dose-f: Zinc high dose fortified; Zn-L-Dose-f: Zinc low dose fortified; Zn-H-f: Zinc high fortified; Zn-L-f: Zinc low fortified; no-s: No supplement; Zn + BCaro-s: zinc plus beta carotene supplement ;BCaro-s: Beta carotene supplement; Zn + MV-s: Zinc plus multivitamin supplement; MV-s: Multivitamin supplement; Zn+VA-s: Zinc plus vitamin A supplement; VA-s: Vitamin A supplement; Zn + VC-s: Zinc plus vitamin C supplement ; VC-s: Vitamin C supplement; Zn + Fe-f: Zinc plus iron fortified; Fe-f: Iron fortified; Zn-s+Fe-f: Zinc supplement plus iron fortified; P-s+Fe-f: Placebo supplement plus iron fortified; SQ-LNS:Zn: Small quantity Lipid Nutrient supplement with zinc; SQ-LNS:No Zn: Small quantity Lipid Nutrient supplement with no zinc; Zn+Fe+FA-s: Zinc plus iron plus folic acid supplement; Fe+FA-s: Iron plus folic acid supplement; Zn in MNP: Micronutrient powder with zinc plus iron; MNP with no Zn: Micronutrient powder with no zinc plus iron; Zn + MN: Zinc plus micronutrient supplement; Zn + Fe + VC-s: Zinc plus iron plus vitamin C supplement.

Figure S6: Effect of additional zinc intervention on serum transferrin receptor concentrations.

Study or Subgroup	zinc Mean	zinc SD	zinc Total	no zinc Mean	no zinc SD	no zinc Total	Weight	Std. Mean Difference IV, Random, 95% CI
0.9mg/d_Becquey 2016 (Zn-s vs no-s)	10.6	9.8	118	10.4	9	127	13.3%	0.02 [-0.23, 0.27]
02.9mg/d_Baqui 2005 (Zn+B2-s vs B2-s)	-0.5	2.7	42	-1.7	3.2	38	9.5%	0.40 [-0.04, 0.85]
02.9mg/d_Baqui 2005 (Zn+Fe+B2-s vs Fe+B2-s)	-2.3	3.1	42	-2	3	41	9.7%	-0.10 [-0.53, 0.33]
07.0mg/d_Becquey 2016 (Zn-s vs no-s)	11	11.1	123	10.3	9	127	13.3%	0.07 [-0.18, 0.32]
10mg/d_Carter 2018 (Zn+MV-s vs MV-s)	1.8	0.9	144	1.7	0.9	147	13.7%	0.11 [-0.12, 0.34]
10mg/d_Carter 2018 (Zn-s vs P-s)	1.8	0.9	151	1.8	0.9	151	13.8%	0.00 [-0.23, 0.23]
10mg/d_Lind 2003 (Zn+Fe-s vs Fe-s)	7.7	1.4	136	6.7	1.3	136	13.4%	0.74 [0.49, 0.98]
10mg/d_Lind 2003 (Zn-s vs P-s)	9.8	1.4	134	9	1.7	143	13.5%	0.51 [0.27, 0.75]
**Total (95% CI)**			**890**			**910**	**100.0%**	**0.22 [0.01, 0.43]**

Heterogeneity: $Tau^2 = 0.07$; $Chi^2 = 33.81$, df = 7 ($P < 0.0001$); $I^2 = 79\%$

Test for overall effect: Z = 2.08 (P = 0.04)

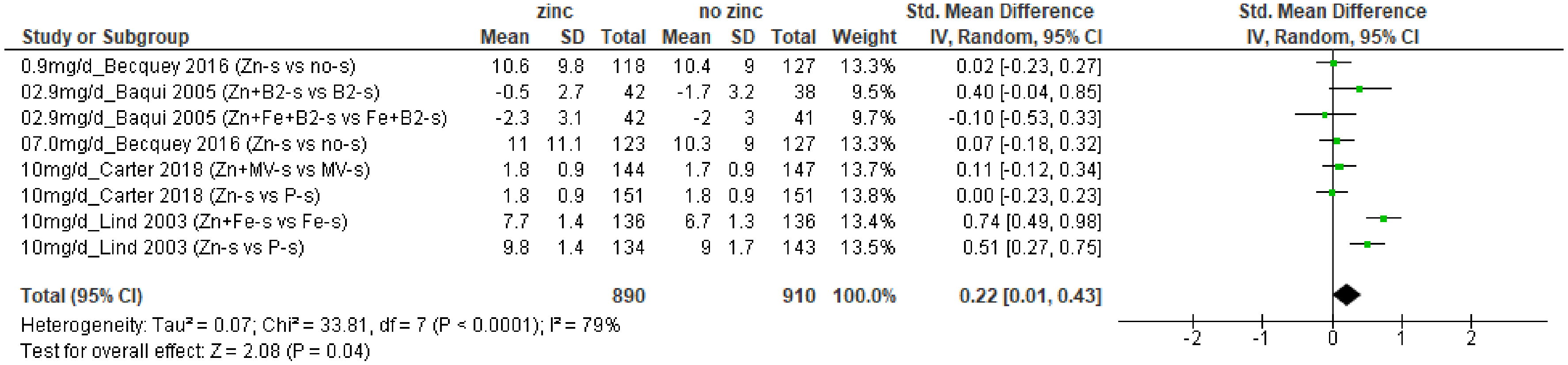

Figure S6 legends: Zn-s: Zinc supplement; P-s: Placebo supplement; Zn + B2-s: Zinc plus vitamin B12 supplement; B2-s: Vitamin B2 supplement; no-s: No supplement

References for studies in supplementary tables

1. Baqui, A.H.; Walker, C.L.; Zaman, K.; El Arifeen, S.; Chowdhury, H.R.; Wahed, M.A.; Black, R.E.; Caulfield, L.E. Weekly iron supplementation does not block increases in serum zinc due to weekly zinc supplementation in Bangladeshi infants. *The Journal of nutrition* **2005**, *135*, 2187-2191, doi:10.1093/jn/135.9.2187.
2. Bertinato, J.; Simpson, J.R.; Sherrard, L.; Taylor, J.; Plouffe, L.J.; Van Dyke, D.; Geleynse, M.; Dam, Y.Y.; Murphy, P.; Knee, C., et al. Zinc supplementation does not alter sensitive biomarkers of copper status in healthy boys. *The Journal of nutrition* **2013**, *143*, 284-289, doi:10.3945/jn.112.171306.
3. Bhandari, N.; Bahl, R.; Taneja, S.; Strand, T.; Molbak, K.; Ulvik, R.J.; Sommerfelt, H.; Bhan, M.K. Substantial reduction in severe diarrheal morbidity by daily zinc supplementation in young north Indian children. *Pediatrics* **2002**, *109*, e86.
4. Brooks, W.A.; Santosham, M.; Naheed, A.; Goswami, D.; Wahed, M.A.; Diener-West, M.; Faruque, A.S.; Black, R.E. Effect of weekly zinc supplements on incidence of pneumonia and diarrhoea in children younger than 2 years in an urban, low-income population in Bangladesh: randomised controlled trial. *Lancet* **2005**, *366*, 999-1004, doi:10.1016/S0140-6736(05)67109-7.
5. Brown, K.H.; Lopez de Romana, D.; Arsenault, J.E.; Peerson, J.M.; Penny, M.E. Comparison of the effects of zinc delivered in a fortified food or a liquid supplement on the growth, morbidity, and plasma zinc concentrations of young Peruvian children. *The American journal of clinical nutrition* **2007**, *85*, 538-547, doi:10.1093/ajcn/85.2.538.
6. Caulfield, L.E.; Zavaleta, N.; Chen, P.; Colombo, J.; Kannass, K. Mineral status of non-anemic Peruvian infants taking an iron and copper syrup with or without zinc from 6 to 18 months of age: a randomized controlled trial. *Nutrition* **2013**, *29*, 1336-1341, doi:10.1016/j.nut.2013.05.023.
7. Hambidge, K.M.; Chavez, M.N.; Brown, R.M.; Walravens, P.A. Zinc nutritional status of young middle-income children and effects of consuming zinc-fortified breakfast cereals. *The American journal of clinical nutrition* **1979**, *32*, 2532-2539, doi:10.1093/ajcn/32.12.2532.
8. Heinig, M.J.; Brown, K.H.; Lonnerdal, B.; Dewey, K.G. Zinc supplementation does not affect growth, morbidity, or motor development of US term breastfed infants at 4-10 mo of age. *The American journal of clinical nutrition* **2006**, *84*, 594-601, doi:10.1093/ajcn/84.3.594.
9. Lind, T.; Lonnerdal, B.; Stenlund, H.; Ismail, D.; Seswandhana, R.; Ekstrom, E.C.; Persson, L.A. A community-based randomized controlled trial of iron and zinc supplementation in Indonesian infants: interactions between iron and zinc. *The American journal of clinical nutrition* **2003**, *77*, 883-890, doi:10.1093/ajcn/77.4.883.
10. Radhakrishna, K.V.; Hemalatha, R.; Geddam, J.J.; Kumar, P.A.; Balakrishna, N.; Shatrugna, V. Effectiveness of zinc supplementation to full term normal infants: a community based double blind, randomized, controlled, clinical trial. *PLoS One* **2013**, *8*, e61486, doi:10.1371/journal.pone.0061486.
11. Rosado, J.L.; Lopez, P.; Kordas, K.; Garcia-Vargas, G.; Ronquillo, D.; Alatorre, J.; Stoltzfus, R.J. Iron and/or zinc supplementation did not reduce blood lead concentrations in children in a randomized, placebo-controlled trial. *The Journal of nutrition* **2006**, *136*, 2378-2383, doi:10.1093/jn/136.9.2378.
12. Ruz, M.; Castillo-Duran, C.; Lara, X.; Codoceo, J.; Rebolledo, A.; Atalah, E. A 14-mo zinc-supplementation trial in apparently healthy Chilean preschool children. *The American journal of clinical nutrition* **1997**, *66*, 1406-1413, doi:10.1093/ajcn/66.6.1406.
13. Sazawal, S.; Malik, P.; Jalla, S.; Krebs, N.; Bhan, M.K.; Black, R.E. Zinc supplementation for four months does not affect plasma copper concentration in infants. *Acta Paediatr* **2004**, *93*, 599-602, doi:10.1111/j.1651-2227.2004.tb18254.x.
14. Tielsch, J.M.; Khatry, S.K.; Stoltzfus, R.J.; Katz, J.; LeClerq, S.C.; Adhikari, R.; Mullany, L.C.; Black, R.; Shresta, S. Effect of daily zinc supplementation on child mortality in southern Nepal: a community-based, cluster randomised, placebo-controlled trial. *Lancet* **2007**, *370*, 1230-1239, doi:10.1016/S0140-6736(07)61539-6.
15. Walravens, P.A.; Hambidge, K.M. Growth of infants fed a zinc supplemented formula. *The American journal of clinical nutrition* **1976**, *29*, 1114-1121, doi:10.1093/ajcn/29.10.1114.
16. Walravens, P.A.; Krebs, N.F.; Hambidge, K.M. Linear growth of low income preschool children receiving a zinc supplement. *The American journal of clinical nutrition* **1983**, *38*, 195-201, doi:10.1093/ajcn/38.2.195.
17. Wuehler, S.E.; Sempertegui, F.; Brown, K.H. Dose-response trial of prophylactic zinc supplements, with or without copper, in young Ecuadorian children at risk of zinc deficiency. *The American journal of clinical nutrition* **2008**, *87*, 723-733, doi:10.1093/ajcn/87.3.723.

18. de Brito, N.J.; Rocha, E.D.; de Araujo Silva, A.; Costa, J.B.; Franca, M.C.; das Gracas Almeida, M.; Brandao-Neto, J. Oral zinc supplementation decreases the serum iron concentration in healthy schoolchildren: a pilot study. *Nutrients* **2014**, *6{Chen, 2008 #1387}*, 3460-3473, doi:10.3390/nu6093460.
19. Bates, C.J.; Evans, P.H.; Dardenne, M.; Prentice, A.; Lunn, P.G.; Northrop-Clewes, C.A.; Hoare, S.; Cole, T.J.; Horan, S.J.; Longman, S.C., et al. A trial of zinc supplementation in young rural Gambian children. *Br J Nutr* **1993**, *69*, 243-255, doi:10.1079/bjn19930026.
20. Abdollahi, M.; Ajami, M.; Abdollahi, Z.; Kalantari, N.; Houshiarrad, A.; Fozouni, F.; Fallahrokni, A.; Mazandarani, F.S. Zinc supplementation is an effective and feasible strategy to prevent growth retardation in 6 to 24 month children: A pragmatic double blind, randomized trial. *Heliyon* **2019**, *5*, e02581, doi:10.1016/j.heliyon.2019.e02581.
21. Alarcon, K.; Kolsteren, P.W.; Prada, A.M.; Chian, A.M.; Velarde, R.E.; Pecho, I.L.; Hoeree, T.F. Effects of separate delivery of zinc or zinc and vitamin A on hemoglobin response, growth, and diarrhea in young Peruvian children receiving iron therapy for anemia. *The American journal of clinical nutrition* **2004**, *80*, 1276-1282, doi:10.1093/ajcn/80.5.1276.
22. Becquey, E.; Ouedraogo, C.T.; Hess, S.Y.; Rouamba, N.; Prince, L.; Ouedraogo, J.B.; Vosti, S.A.; Brown, K.H. Comparison of Preventive and Therapeutic Zinc Supplementation in Young Children in Burkina Faso: A Cluster-Randomized, Community-Based Trial. *The Journal of nutrition* **2016**, *146*, 2058-2066, doi:10.3945/jn.116.230128.
23. Berger, J.; Ninh, N.X.; Khan, N.C.; Nhien, N.V.; Lien, D.K.; Trung, N.Q.; Khoi, H.H. Efficacy of combined iron and zinc supplementation on micronutrient status and growth in Vietnamese infants. *European journal of clinical nutrition* **2006**, *60*, 443-454, doi:10.1038/sj.ejcn.1602336.
24. Carter, R.C.; Kupka, R.; Manji, K.; McDonald, C.M.; Aboud, S.; Erhardt, J.G.; Gosselin, K.; Kisenge, R.; Liu, E.; Fawzi, W., et al. Zinc and multivitamin supplementation have contrasting effects on infant iron status: a randomized, double-blind, placebo-controlled clinical trial. *European journal of clinical nutrition* **2018**, *72*, 130-135, doi:10.1038/ejcn.2017.138.
25. Dijkhuizen, M.A.; Wieringa, F.T.; West, C.E.; Martuti, S.; Muhilal. Effects of iron and zinc supplementation in Indonesian infants on micronutrient status and growth. *The Journal of nutrition* **2001**, *131*, 2860-2865, doi:10.1093/jn/131.11.2860.
26. Fahmida, U.; Rumawas, J.S.; Utomo, B.; Patmonodewo, S.; Schultink, W. Zinc-iron, but not zinc-alone supplementation, increased linear growth of stunted infants with low haemoglobin. *Asia Pac J Clin Nutr* **2007**, *16*, 301-309.
27. Lopez de Romana, D.; Salazar, M.; Hambidge, K.M.; Penny, M.E.; Peerson, J.M.; Krebs, N.F.; Brown, K.H. Longitudinal measurements of zinc absorption in Peruvian children consuming wheat products fortified with iron only or iron and 1 of 2 amounts of zinc. *The American journal of clinical nutrition* **2005**, *81*, 637-647, doi:10.1093/ajcn/81.3.637.
28. Rosado, J.L.; Lopez, P.; Munoz, E.; Martinez, H.; Allen, L.H. Zinc supplementation reduced morbidity, but neither zinc nor iron supplementation affected growth or body composition of Mexican preschoolers. *The American journal of clinical nutrition* **1997**, *65*, 13-19, doi:10.1093/ajcn/65.1.13.
29. Soofi, S.; Cousens, S.; Iqbal, S.P.; Akhund, T.; Khan, J.; Ahmed, I.; Zaidi, A.K.; Bhutta, Z.A. Effect of provision of daily zinc and iron with several micronutrients on growth and morbidity among young children in Pakistan: a cluster-randomised trial. *Lancet* **2013**, *382*, 29-40, doi:10.1016/S0140-6736(13)60437-7.
30. Tielsch, J.M.; Khatry, S.K.; Stoltzfus, R.J.; Katz, J.; LeClerq, S.C.; Adhikari, R.; Mullany, L.C.; Shresta, S.; Black, R.E. Effect of routine prophylactic supplementation with iron and folic acid on preschool child mortality in southern Nepal: community-based, cluster-randomised, placebo-controlled trial. *Lancet* **2006**, *367*, 144-152, doi:10.1016/S0140-6736(06)67963-4.
31. Veenemans, J.; Milligan, P.; Prentice, A.M.; Schouten, L.R.; Inja, N.; van der Heijden, A.C.; de Boer, L.C.; Jansen, E.J.; Koopmans, A.E.; Enthoven, W.T., et al. Effect of supplementation with zinc and other micronutrients on malaria in Tanzanian children: a randomised trial. *PLoS Med* **2011**, *8*, e1001125, doi:10.1371/journal.pmed.1001125.
32. Wasantwisut, E.; Winichagoon, P.; Chitchumroonchokchai, C.; Yamborisut, U.; Boonpraderm, A.; Pongcharoen, T.; Sranacharoenpong, K.; Russameesopaphorn, W. Iron and zinc supplementation improved iron and zinc status, but not physical growth, of apparently healthy, breast-fed infants in rural communities of northeast Thailand. *The Journal of nutrition* **2006**, *136*, 2405-2411, doi:10.1093/jn/136.9.2405.

33. Wieringa, F.T.; Dijkhuizen, M.A.; West, C.E.; Thurnham, D.I.; Muhilal; Van der Meer, J.W. Redistribution of vitamin A after iron supplementation in Indonesian infants. *The American journal of clinical nutrition* **2003**, *77*, 651-657, doi:10.1093/ajcn/77.3.651.
34. Zlotkin, S.; Arthur, P.; Schauer, C.; Antwi, K.Y.; Yeung, G.; Piekarz, A. Home-fortification with iron and zinc sprinkles or iron sprinkles alone successfully treats anemia in infants and young children. *The Journal of nutrition* **2003**, *133*, 1075-1080, doi:10.1093/jn/133.4.1075.
35. Hess, S.Y.; Abbeddou, S.; Jimenez, E.Y.; Some, J.W.; Vosti, S.A.; Ouedraogo, Z.P.; Guissou, R.M.; Ouedraogo, J.B.; Brown, K.H. Small-quantity lipid-based nutrient supplements, regardless of their zinc content, increase growth and reduce the prevalence of stunting and wasting in young burkinabe children: a cluster-randomized trial. *PLoS One* **2015**, *10*, e0122242, doi:10.1371/journal.pone.0122242.
36. Olney, D.K.; Pollitt, E.; Kariger, P.K.; Khalfan, S.S.; Ali, N.S.; Tielsch, J.M.; Sazawal, S.; Black, R.; Allen, L.H.; Stoltzfus, R.J. Combined iron and folic acid supplementation with or without zinc reduces time to walking unassisted among Zanzibari infants 5- to 11-mo old. *The Journal of nutrition* **2006**, *136*, 2427-2434, doi:10.1093/jn/136.9.2427.
37. Owusu-Agyei, S.; Newton, S.; Mahama, E.; Febir, L.G.; Ali, M.; Adjei, K.; Tchum, K.; Alhassan, L.; Moleah, T.; Tanumihardjo, S.A. Impact of vitamin A with zinc supplementation on malaria morbidity in Ghana. *Nutr J* **2013**, *12*, 131, doi:10.1186/1475-2891-12-131.
38. Penny, M.E.; Marin, R.M.; Duran, A.; Peerson, J.M.; Lanata, C.F.; Lonnerdal, B.; Black, R.E.; Brown, K.H. Randomized controlled trial of the effect of daily supplementation with zinc or multiple micronutrients on the morbidity, growth, and micronutrient status of young Peruvian children. *The American journal of clinical nutrition* **2004**, *79*, 457-465, doi:10.1093/ajcn/79.3.457.
39. Richard, S.A.; Zavaleta, N.; Caulfield, L.E.; Black, R.E.; Witzig, R.S.; Shankar, A.H. Zinc and iron supplementation and malaria, diarrhea, and respiratory infections in children in the Peruvian Amazon. *Am J Trop Med Hyg* **2006**, *75*, 126-132, doi:10.4269/ajtmh.2006.75.1.0750126.
40. Sazawal, S.; Dhingra, U.; Dhingra, P.; Dutta, A.; Deb, S.; Kumar, J.; Devi, P.; Prakash, A. Efficacy of high zinc biofortified wheat in improvement of micronutrient status, and prevention of morbidity among preschool children and women - a double masked, randomized, controlled trial. *Nutr J* **2018**, *17*, 86, doi:10.1186/s12937-018-0391-5.
41. Shankar, A.H.; Genton, B.; Baisor, M.; Paino, J.; Tamja, S.; Adiguma, T.; Wu, L.; Rare, L.; Bannon, D.; Tielsch, J.M., et al. The influence of zinc supplementation on morbidity due to Plasmodium falciparum: a randomized trial in preschool children in Papua New Guinea. *Am J Trop Med Hyg* **2000**, *62*, 663-669, doi:10.4269/ajtmh.2000.62.663.
42. Smith, J.C.; Makdani, D.; Hegar, A.; Rao, D.; Douglass, L.W. Vitamin A and zinc supplementation of preschool children. *J Am Coll Nutr* **1999**, *18*, 213-222, doi:10.1080/07315724.1999.10718854.
43. Taneja, S.; Bhandari, N.; Rongsen-Chandola, T.; Mahalanabis, D.; Fontaine, O.; Bhan, M.K. Effect of zinc supplementation on morbidity and growth in hospital-born, low-birth-weight infants. *The American journal of clinical nutrition* **2009**, *90*, 385-391, doi:10.3945/ajcn.2009.27707.
44. Chimhashu, T.; Malan, L.; Baumgartner, J.; van Jaarsveld, P.J.; Galetti, V.; Moretti, D.; Smuts, C.M.; Zimmermann, M.B. Sensitivity of fatty acid desaturation and elongation to plasma zinc concentration: a randomised controlled trial in Beninese children. *Br J Nutr* **2018**, *119*, 610-619, doi:10.1017/S000711451700366X.
